# Molecular resistome profiling of multidrug-resistant *K. pneumoniae* from a tertiary-care hospital in North India

**DOI:** 10.6026/973206300221295

**Published:** 2026-03-31

**Authors:** Rashmi, Vimala Venkatesh, Sheetal Verma, Upma Singh

**Affiliations:** 1 Department of Microbiology, Faculty of Medicine, King George's Medical University, Lucknow, India

**Keywords:** Multi-drug resistance (MDR), antimicrobial resistance (AMR), extended-spectrum beta-lactamase (ESBLs), carbapenemase, super-resistome, carbapenem-resistant, *K. pneumoniae*

## Abstract

*K. pneumoniae* is a major opportunistic pathogen and an important cause of multidrug-resistant gram-negative infections, driven largely by the spread of ESBL and carbapenemase enzymes. Therefore, it is of interest to analyse the phenotypic resistance patterns and molecular resistome of 100 non-duplicate clinical isolates collected from a tertiary care hospital in North India using CLSI- guided susceptibility testing, mCIM/eCIM assays and PCR detection of resistance genes. Overall, 51% of isolates were MDR, with high resistance to cephalosporins, fluoroquinolones and penicillins and the highest carbapenem resistance was observed for ertapenem (57%). Carbapenem- resistant isolates comprised 40%, of which 70% produced metallo-β-lactamases and 10% serine β-lactamases, while ESBL genes (blaCTX-M-1 54%, blaSHV 50%, blaTEM 28%) and carbapenemases (blaNDM 22%, blaOXA-48 23%) were frequently detected, including ESBL-carbapenemase co-harbouring in 35% and a "super-resistome" in 2%. Thus, we show the urgent need for sustained molecular surveillance, strict infection control and strengthened antimicrobial stewardship to curb the dissemination of highly resistant *K. pneumoniae*.

## Background:

The continuous emergence and threat of multidrug-resistant (MDR) Gram-negative bacilli, specifically *K. pneumoniae*, has led to renewed research interest. *K. pneumoniae* is a gram-negative, non-motile, encapsulated opportunistic pathogen belonging to the Enterobacteriaceae family [1]. It widely exists in the environment, including soil, water and plants and commonly colonizes human mucosal surfaces, causing several opportunistic infections, including urinary tract infections, pneumonia, post-traumatic infections, endocarditis and sepsis, particularly in patients with compromised immune systems or prolonged hospitalization; however, it is often harmless in healthy individuals [2]. Potential to acquire both community and healthcare settings and integrated antibiotic resistance, has made *K. pneumoniae* a life-threatening global health concern. This bacterium is a significant contributor to the growing trend of drug-resistant infections, according to the European Antimicrobial Resistance Surveillance Network [3]. The global burden of antimicrobial resistance (AMR) in *K. pneumoniae* has risen drastically in recent decades, exhibiting resistance to multiple classes of antibiotics, including β-lactams, fluoroquinolones and aminoglycosides. A major concern is the emergence of strains producing extended-spectrum β-lactamases (ESBLs) and carbapenemases, which severely limit treatment options [4, 5-6]. The World Health Organisation (WHO) also reported that ESBL-driven *K. pneumoniae* has become predominant in several regions worldwide, with increasing resistance even in community- acquired infections. The rise in resistance has been driven by horizontal gene transfer, plasmid-mediated mechanisms and the rapid dissemination of high-risk clonal lineages [7]. *K. pneumoniae* exhibits remarkable genomic diversity, often harbouring multiple resistance genes, especially β-lactamase (bla) gene on mobile genetic elements such as plasmids and transposons collectively called the "mobilome". This "mobilome," a network of transferable elements carrying antibiotic resistance genes (ARGs), works in tandem with the "resistome" which encompasses the complete set of resistance genes in the genome, to drive the evolution and spread of resistance [7, 8]. Surprisingly, strains with multiple plasmids or composite plasmids significantly lead to MDR, extensively drug resistance (XDR) and, in rare cases, pan-drug resistance (PDR) [9]. Furthermore, the extensive use of carbapenems has led to the development of plasmid-mediated carbapenemases capable of breaking down all β-lactam antibiotics, including these critical last-resort treatments [10]. The increasing reliance on carbapenems as last-resort antibiotics has further accelerated the emergence of carbapenem-resistant *K. pneumoniae* (CRKP) over the past two decades. The co-existence of resistome-driven resistance and virulence factors, particularly in *K. pneumoniae* strains, presents additional clinical challenges, particularly in the case of serious infections with limited treatment options. Given the significant public health concern, *K. pneumoniae* has been recognised as a potent pathogen by the WHO, CDC and other international health bodies [11]. Therefore, it is of interest to investigate the genetic and enzymatic mechanisms that enable antimicrobial resistance (AMR) in *K. pneumoniae*. Further, the role of resistance genes in the emergence of multidrug resistance (MDR) in clinical settings is of interest.

## Materials and Methods:

## Bacterial isolation and identification:

A total of 100 non-duplicate *K. pneumoniae* strains were collected from the Department of Microbiology, Bacteriology Unit, King George's Medical University, Lucknow, India. All strains were isolated from various clinical samples, including blood, urine, pus, respiratory fluids, body fluids and CSF. All collected samples were initially cultured and incubated to promote bacterial growth, followed by species identification using MALDI-TOF mass spectrometry. These isolates were stored in 50% glycerol stock at -80°C and revived by subculturing on MacConkey agar for further use.

## Antimicrobial susceptibility profiling:

Antimicrobial susceptibility profiling of all identified *K. pneumoniae* strains was performed by Kirby-Bauer disk diffusion method with respective antibiotics. However, Norfloxacin (NX), Nitrofurantoin (NIT) and Fosfomycin (FO) were only tested for urine samples, along with all antibiotics. The results were interpreted as per Clinical and Laboratory Standards Institute (CLSI) guidelines [12]. Accordingly, isolates that exhibited resistance to at least one antibiotic in three or more distinct antimicrobial classes were categorized as multidrug-resistant (MDR) [13].

## Phenotypic detection of carbapenem-resistant *K. pneumoniae* (CRKP):

Carbapenemase activity in CRKP isolates was determined through the modified carbapenem inactivation method (mCIM) and EDTA-carbapenem inactivation method (eCIM) according to CLSI standard protocols [12].

## Molecular characterisation:

Genomic DNA was extracted from bacterial isolates using the boiling method followed by conventional PCR, targeting resistome and antimicrobial resistance (AMR) genes, i.e., β-lactamase genes Class A- bla (SHV, TEM, VEB, PER, GES and CTXM-1), as well as Carbapenemases producing genes bla (KPC, IMP, VIM, OXA-48 and NDM). Subsequently, the isolates were screened for the presence of Ambler class C (AmpC) β-lactamase and aminoglycoside-modifying enzyme resistance genes using multiplex PCR targeting bla (MOXM, CITM, ACCM and FOXM) and (aac3'-1a, acc3'-2a, aph3'-1a and ant2"-1a) (Table 1 - see PDF).

## Statistical analysis:

All obtained data were maintained in Microsoft Excel and analyzed using MedCalc statistical software (MedCalc Software Ltd., Version 23.0.9). The "Comparison of Means Calculator" was used to evaluate differences between data sets (https://www.medcalc.org/calc/comparison_ of_means.php). Phenotypic and genotypic characterization of *K. pneumoniae* isolates was correlated using a chi-square test. A p-value of less than 0.05 was considered statistically significant.

## Results and Discussion:

Among 100 isolates of *K. pneumoniae* obtained from a variety of clinical specimens. These included blood samples (20%), urine samples (31%), respiratory fluids (18%), exudates such as pus, drains, swabs, or abscesses (18%), body fluids (9%) and cerebrospinal fluid (CSF) (4%). The clinical specimens were collected from multiple wards and departments within the hospital, which included cardiology (1%), emergency/trauma (3%), medicine (7%), nephrology (1%), orthopedic surgery (1%), pediatrics (3%), plastic surgery (2%), gynaecology (4%), respiratory medicine (3%), surgical gastroenterology (2%), trauma surgery (1%), urology (5%), intensive care units (14%) and general wards (53%). The average age of patients with *K. pneumoniae* infections was 41.90 ± 20.85 years. Additionally, it was noted that *K. pneumoniae* infections were more frequent in male (59%) patients in comparison to female (41%) patients, respectively. All the identified *K. pneumoniae* isolates were tested for antimicrobial susceptibility profiling and showed a high burden of resistance to various classes of antibiotics. Cephalosporins and fluoroquinolones displayed widespread and statistically significant resistance (p < 0.05), limiting their therapeutic value. Interestingly, aminoglycosides, including amikacin (AMK), gentamycin (GEN) and tobramycin (TOB), showed overall 50% resistance among all isolates. In carbapenems, ertapenem (ETP) showed higher resistance, while imipenem (IMP) and meropenem (MRP) demonstrated significantly better activity (p = 0.0003 and 0.0278, respectively). Ampicillin (AMP) showed 100% intrinsic resistance, whereas piperacillin-tazobactam (PIT) and amoxicillin-clavulanate (AMC) had moderate resistance. Tetracycline (TE) showed lower resistance statistically significant (p = 0.0164). Notably, indicating strong potential for empirical use (Table 2 - see PDF). 51% of the total isolates demonstrated multi-drug resistance (MDR) (Figure 1 - see PDF). A total of 40% isolates showed phenotypic resistance to at least one carbapenem antibiotic, including IMP, MRP and ETP, which were selected for the Carbapenemase production test. These isolates were identified as Carbapenem-resistant *K. pneumoniae* (CRKP). Out of the 40% of CRKP isolates examined, 28 (70%) were further subjected to both the mCIM and eCIM tests and demonstrated positive results, indicating the significant producers of Metallo β-lactamase (MBL) belonging to Ambler class B. Furthermore, 4 isolates (10%) that tested positive exclusively for the mCIM test were found to produce Serine β-lactamase (SBL) enzymes from Ambler classes A and D. Additionally, 8 isolates (20%) were determined not to produce any Carbapenemase enzymes (Figure 2 - see PDF) and interpretation shown in (Table 3 - see PDF).

The electrophoretic band patterns corresponded to the expected amplicon sizes for each target gene, confirming successful amplification and presence of the respective resistance determinants in isolates (Figure 3 - see PDF). Molecular analysis has revealed a substantial prevalence of ESBL genes, namely blaCTXM-1, blaSHV and blaTEM, were found within the examined isolates, with respective frequencies of 54%, 50% and 28%. These findings suggest a significant level of resistance to β-lactam antibiotics. In addition, Carbapenemase genes, such as blaNDM and blaOXA-48, are comparatively less prevalent; nonetheless, they significantly contribute to antibiotic resistance, with frequencies of 22% and 23%, respectively. Conversely, the prevalence of the AmpC β-lactamase gene blaCITM and the AME genes was significantly low in this dataset (Figure 4 - see PDF). Molecular testing identified the existence of a resistome; analysis of all *K. pneumoniae* isolates revealed significant findings regarding the co-occurrence of AMR genes. Interestingly, 2% of the total isolates potentially developed a "super resistome," characterized by the presence of key AMR genes, including ESBLs, Carbapenemases, AmpC β-lactamases and AMEs. Meanwhile, the findings revealed that 35% of isolates co-harboured both ESBL and carbapenemase-producing genes, indicating a high incidence of the MDR phenotype. The occurrence of this diverse resistome profile in specific isolates highlights a unique clinical profile of the study subject, which may lead to resistance to a wide range of conventional drugs. The detailed distribution of these co-harboured resistance determinants is summarised in (Table 4 - see PDF). The molecular analysis of AMR mechanisms indicates that ESBL group represents the most prevalent resistance mechanism, affecting 77% of the total isolates examined. ESBL genes confer resistance to a broad spectrum of beta-lactam antibiotics, including penicillins and cephalosporins. Additionally, carbapenemase enzymes, which degrade carbapenems, were identified in 38% of the total isolates. Furthermore, AmpC beta-lactamase and AME were detected in 11% and 10% of the total isolates, respectively (Figure 5 - see PDF).

*K. pneumoniae* remains a major nosocomial pathogen and the accelerating spread of antimicrobial resistance (AMR) continues to erode effective treatment options in tertiary-care settings [22-23]. In our cohort, multidrug resistance (MDR) was frequent and was primarily driven by β-lactam resistance mechanisms, particularly extended-spectrum β-lactamases (ESBLs) and carbapenemases [22, 23 24-25]. This pattern is consistent with recent PubMed-indexed surveillance and genomic studies identifying *K. pneumoniae* as a leading cause of difficult-to-treat Gram-negative infections [24]. ESBL genes were the most common resistance determinants in our isolates, dominated by blaCTX-M-1 together with blaSHV and blaTEM [25]. Contemporary studies consistently report CTX-M enzymes as the predominant ESBL family, often co-existing with SHV- and TEM-type β-lactamases, explaining the high phenotypic resistance observed to third-generation cephalosporins [23, 24-25]. Carbapenem non-susceptibility-particularly to ertapenem was substantial and phenotypic carbapenemase activity was detected among CRKP isolates [24, 25, 26- 27]. Recent regional studies demonstrate a high prevalence of carbapenem-resistant *K. pneumoniae*, with co-dominance of blaNDM and blaOXA-48-like genes [25, 26, 27-28]. OXA-48 like producers may be under-recognised by routine phenotypic assays, facilitating silent dissemination [28]. A key observation was the accumulation of multiple resistance mechanisms within single isolates, including ESBL + carbapenemase co-carriage and a subset with expanded resistomes (ESBL + carbapenemase + AmpC + AME genes). This convergence is often driven by mobile genetic elements and clonal expansion and is associated with reduced treatment options and higher risk of therapeutic failure [26, 27, 28-29]. Given the dominance of NDM and OXA-48 like enzymes and diagnostic challenges with some variants, routine integration of mechanism-oriented diagnostics is critical to optimize therapy, support infection-control actions and strengthen stewardship [28]. The combined phenotypic and molecular approach improved resistance characterization and enabled detection of complex co-carriage patterns [27, 28-29]. However, the single-center design limits generalizability and whole-genome sequencing would provide better resolution of transmission pathways. Future studies integrating genomic epidemiology with patient outcomes are needed [26, 27, 28-29]. A key finding was the co-existence of multiple resistance mechanisms. More than one-third of isolates carried both ESBL and carbapenemase genes and a small subset exhibited a "super-resistome" comprising ESBL, carbapenemase, AmpC and aminoglycoside-modifying enzyme genes. These resistance patterns have important clinical implications. The dominance of NDM and OXA-48-like enzymes limits the effectiveness of several newer β-lactam/β-lactamase inhibitor combinations and requires mechanism-based therapy, often including combination regimens in severe infections. This highlights the need for integrating molecular diagnostics into routine clinical practice. The combined use of phenotypic and molecular methods improved resistance characterization and supported targeted infection control and stewardship. However, the single-centre design, lack of whole-genome sequencing and absence of clinical outcome data limit generalizability. Future studies integrating genomic surveillance and patient outcomes are needed.

## Conclusion:

Multidrug-resistant *K. pneumoniae* was highly prevalent in the studied tertiary-care hospital, driven primarily by ESBL and carbapenemase mechanisms, and frequent co-harbouring of these genes, including NDM and OXA-48, highlights extensive plasmid-mediated dissemination of resistance determinants. The identification of isolates carrying a complex "super-resistome," encompassing multiple resistance mechanisms across different antimicrobial classes, highlights an alarming genetic convergence that severely restricts available therapeutic options and increases the risk of treatment failure, prolonged hospital stays and higher morbidity and mortality. Routine integration of molecular diagnostics with phenotypic testing is essential for effective surveillance, infection control and antimicrobial stewardship.

## Advancement of knowledge:

Provides combined phenotypic and extended PCR-based resistome profiling of clinical *K. pneumoniae* isolates from North India. Demonstrate high ESBL-carbapenemase co-carriage and documents emergence of rare multi-mechanism "super-resistome" isolates. Supports integration of molecular resistance screening with routine diagnostics for improve antimicrobial stewardship and surveillance.

## Funding source:

Ms. Rashmi is supported by the Senior Research Fellowship (SRF) from the Council of Scientific and Industrial Research, Government of India. The funders did not participate in the study design, data collection and analysis, decision-making regarding publication, or manuscript preparation.

## Author contribution:

VV: Designed the research, supervised it, provided resources and reviewed and edited the manuscript. R: conducted all experiments, data analysis and wrote the manuscript (drafting, review and editing). SV: monitored experiments. US: manuscript review and editing assistance.
